# The role and underlying mechanisms of Qi Gong Wan in enhancing the endometrial receptivity of a rat model with polycystic ovary syndrome

**DOI:** 10.3389/frph.2025.1733583

**Published:** 2026-03-03

**Authors:** Chun Ding, Qinhua Li, Yiwen Deng, Yuhan Liu, Lei Liu, ChunYu Cao, Siwei Li, Tingting Zheng, Liangqun Xie, Jing Zhao, Hong Ye, Junkui Li

**Affiliations:** 1 Institute of Obstetrics and Gynecology, The First College of Clinical Medical Science, China Three Gorges University, Yichang, China; 2 Central Laboratory, The First College of Clinical Medical Science, China Three Gorges University & Yichang Central People’s Hospital, Yichang, China; 3 Hubei Key Laboratory of Tumor Microenvironment and Immunotherapy, Yichang, China; 4 Department of Obstetrics and Gynecology, The 983rd Hospital of Joint Service Support Force of Chinese PLA, Tianjin, China

**Keywords:** endometrial receptivity, herb, infertility, insulin resistance, polycystic ovary syndrome, Qi Gong Wan

## Abstract

**Background:**

Qi Gong Wan (QGW) is a herbal formula which is used for treating infertility associated with polycystic ovary syndrome (PCOS). However, the mechanism of action remains unclear. This study aimed to investigate whether QGW enhances endometrial receptivity in a PCOS with insulin resistance (PCOS-IR) rat model and to explore the underlying molecular mechanisms and primary active constituents.

**Materials and methods:**

A PCOS with insulin resistance (IR) rat model was established using dehydroepiandrosterone (DHEA) and a high-fat diet. Rats were treated with QGW or metformin as a positive control. Network pharmacology and molecular docking were used to identify potential drug-disease targets and active components. Endometrial receptivity was evaluated by assessing key markers—including HOXA10, HOXA11, ITGβ3, LIF, GLUT4, and IGFBP1—using histological examination, scanning electron microscopy (to observe pinopode formation), quantitative real-time PCR, Western blot, and immunohistochemistry. SiRNA-mediated knockdown of Hoxa11 was employed to validate its functional role. Network pharmacology and molecular docking techniques were applied to identify potential drug–disease targets and active constituents.

**Results:**

QGW significantly restored regular estrous cycles, reduced testosterone, fasting insulin, and HOMA-IR levels, and improved ovarian morphology in PCOS-IR rats. Network pharmacological analysis identifies HOXA10, HOXA11, and IGFBP1 as core targets. Molecular docking studies demonstrate that wogonin exhibits strong binding affinity with these targets. QGW upregulates the expression of endometrial receptivity markers (HOXA10, HOXA11, ITGβ3, LIF, GLUT4, IGF1) while downregulating IGFBP1 and IL-6 levels. Additionally, Qigui Wenjing Formula promotes pinopode formation and normalizes estrogen/progesterone receptor expression. When Hoxa11 gene expression is suppressed, this formula can reverse the consequent decline in receptivity-related gene expression.

**Conclusions:**

QGW is capable of enhancing the expression of genes related to endometrial receptivity in PCOS model rats and increasing the number of pinopodes, thereby improving the endometrial receptivity in PCOS rats. The results suggest that QGW may improve endometrial receptivity potentially through upregulation of Hoxa11 accompanied by increased Itgβ3 and decreased Igfbp1. However, whether Hoxa11 directly binds to the promoter regions of relevant genes requires further validation. Network pharmacology and molecular docking suggest that wogonin may be an active constituent with considerable potential, though its specific contribution requires further validation through in vivo and in vitro functional experiments.

## Graphical Abstract

**Figure:**
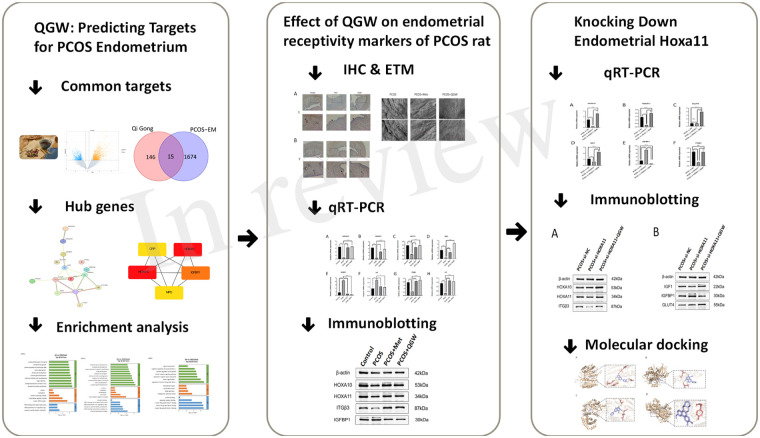
The graphical abstract outlines the mechanistic study of Qigong Wan (QGW) for treating polycystic ovary syndrome (PCOS) endometrium.

## 1 Introduction

PCOS is a common reproductive endocrine metabolic disorder characterized by hyperandrogenism or related clinical manifestations (such as acne, hirsutism), polycystic ovarian morphology, and ovulatory dysfunction. These pathological changes collectively lead to menstrual irregularities, infertility, and other health issues, often accompanied by obesity and IR, severely affecting the reproductive and metabolic functions of patients (1). Women of reproductive age with PCOS primarily exhibit ovulatory dysfunction, and even after adjusting lifestyle, correcting metabolic disorders, and ovulation, the clinical pregnancy rate is only about 35%–40% (2, 3). In the process of *in vitro* fertilization-embryo transfer (IVF-ET) assistance, PCOS patients do not see an increased pregnancy rate even with the transfer of high-quality embryos (4, 5), indicating that the fertility of PCOS patients is reduced, not only due to ovulatory disorders but also related to decreased endometrial receptivity. Studies have shown that the expression of endometrial receptivity-related molecular markers such as Homeobox A (HOXA) 10, HOXA11, Leukemia Inhibitory Factor (LIF), Integrin Beta 3 (ITGβ3), and Glucose Transporter 4 (GLUT4) is reduced in the endometrium of PCOS patients, indicating that reduced endometrial receptivity is one of the factors leading to infertility in PCOS patients (6–8). Metformin is the most common insulin-sensitizing drug used for PCOS with IR, which can reduce weight, improve menstruation, and improve endometrial receptivity, but side effects such as diarrhea and abdominal discomfort are also common (9, 10). Therefore, it is necessary to explore the use of drugs with fewer side effects and reliable treatment efficiency.

In ancient Chinese medical literature, the term “PCOS” is not explicitly mentioned. However, PCOS manifests clinically with features such as oligomenorrhea, amenorrhea, obesity, and infertility, which are consistent with the phlegm-dampness phenotype of infertility treated with QGW in the Qing Dynasty medical compendium “*Yi Fang Ji Jie.*” The main components of QGW are Ban Xia {*Pinellia ternata* (Thunb.) Makino}, Cang Zhu (*Atractylodes lancea* (Thunb.) DC*.*), Xiang Fu (*Cyperus rotundus* L*.*), Fu Ling {*Poria cocos* (Schw.) Wolf.}, Chen Pi (*Tangerine peel*), Chuan Xiong (*Conioselinum anthriscoides ‘Chuanxiong’.*), and Shen Qu *(Massa Medicata Fermentata*).The plant names have been verified against http://mpns.kew.org on December 10, 2024. To date, QGW has been widely used to treat infertile patients with PCOS (11, 12). QGW has demonstrated significant therapeutic efficacy in the clinical treatment of PCOS. It has been proven to increase pregnancy rates among infertile PCOS patients, alleviate clinical symptoms, and enhance the quality and developmental potential of oocytes (12, 13). QGW can regulate ovarian hormone secretion in PCOS patients, promote follicular development and ovulation (14), and inhibit granulosa cell apoptosis, thereby improving oocyte quality and embryonic developmental potential (13). In a PCOS-IR mouse model, QGW reduced the average diameter and total area of adipocytes (15). QGW can also alleviate the chronic low-grade inflammatory state of PCOS and improve the pathological state of PCOS-IR (15). QGW is capable of increasing endometrial thickness in infertile PCOS patients, reducing the uterine artery resistance index (RI), and enhancing pregnancy rates (11, 16). Ovulatory dysfunction is one of the factors associated with infertility in PCOS, and abnormal endometrial receptivity is also a factor contributing to infertility in PCOS patients. However, it is still unclear whether QGW improves pregnancy rates in PCOS by enhancing endometrial receptivity. This study combines animal experiments and network pharmacology to clarify the role of QGW in improving endometrial receptivity and preliminarily explores its mechanism of action.

## 2 Methods and materials

### 2.1 Experimental animals

A total of 65 specific pathogen-free (SPF) grade female Sprague-Dawley (SD) rats, aged 3 weeks and weighing between 52 and 66 g, were purchased and housed at the Animal Experiment Center of China Three Gorges University (Animal Certificate No. 42010200008980, No. 42010200009387). The rats had free access to water and food and were maintained under a 12-hour light/dark cycle. After a one-week acclimation period without intervention, the rats were used to establish a PCOS model. The study involving experimental animals have been conducted in accordance with the ARRIVE guidelines, the U.K. Animals (Scientific Procedures) Act, 1986 and associated guidelines, EU Directive 2010/63/EU for animal experiments, and the National Research Council's Guide for the Care and Use of Laboratory Animals. Ethical approval for this research was granted by the Animal Welfare Ethics Committee of China Three Gorges University (Approval Number: 2020080X).

### 2.2 Establishment and identification of PCOS-IR rat model

Establishment of PCOS Rat Model: The model (PCOS) group of rats received continuous subcutaneous injections on the back of the neck of DHEA (0.06 mg/(g*d); Aladdin Industrial Corporation, Shanghai) and sesame oil (0.1 ml/rat; Shanghai Macklin Biochemical Technology Co., Ltd.), once daily for 24 consecutive days, along with a high-fat diet (consisting of 56% basic feed, 12% lard, 2% soybean oil, 5% egg yolk powder, 20% sugar, 3% milk powder, 1.5% cholesterol, 0.5% sodium deoxycholate; Baituo Experimental Feed Business Department, Hongshan District, Wuhan). The control (Control) group received the same dose of sesame oil by subcutaneous injection on the back of the neck, once daily for 24 consecutive days, and were given a regular diet. Daily vaginal smears were used to monitor the estrous cycle, and hematoxylin and eosin (HE) staining was used to observe morphological changes in the ovaries. Fasting blood glucose, insulin, and sex hormone levels were assessed to evaluate the success of the PCOS model establishment.

### 2.3 Drug treatment

After successful modeling, the rats were further divided into three groups: the model (PCOS) group, the model plus metformin (PCOS + Met) group, and the model plus QGW (PCOS + QGW) group. That is, the PCOS group received intragastric injection of physiological saline, the PCOS + Met group received intragastric injection of metformin hydrochloride (500 mg/kg/d, Merck Pharmaceuticals), which the dose of metformin is the same as the dose used in PCOS model in rats in other reported studies (24), and the PCOS + QGW group received intragastric administration of QGW prescription, which originates from the Qing Dynasty's “*Yi Fang Ji Jie*”. Containing 12 g of *Pinellia ternata* (Thunb.) Makino*; *Batch Number 240101, 8 g of *Atractylodes lancea* (Thunb.) DC.; Batch Number 241001, 16 g of *Cyperus rotundus* L.; Batch Number 240402*,* 12 g of *Poria cocos* (Schw.) Wolf.; Batch Number 240801, 8 g of *Massa medicata fermentata*; Batch Number 231001, 8 g of *Citrus reticulata Blanco*; Batch Number 231001), and 8 g of *Conioselinum anthriscoides ‘Chuanxiong’.* Batch Number 240301; these herbal granules were purchased from China National Medicines Corporation Zhonglian Pharmaceutical Co., Ltd. Prepared according to the aforementioned ratio, a single dose of QGW granules weighs 12.66 g, and a 70 kg adult takes this amount daily. To translate the daily human dose to a rat's dose, Rat dose = 6.3 × Human dose/Human weight × Rat weight. The low dose of QGW = 6.3 × (*12.66 g*/70 kg) = 1.1394 g/kg, with the doses in the ratio of 1:2:4 for low, medium, and high doses, respectively. Based on the results of preliminary experiments, both the medium dose and high dose of QGW were able to significantly reduce fasting insulin and Home-IR levels. We selected the high dosage for intragastric administration, with equal volumes for each group, administered once daily, divided into two administrations in the morning and evening, for 10 consecutive days.

The rats were anesthetized with an intraperitoneal injection of 3% pentobarbital sodium (100 mg/kg), and blood was collected from the abdominal aorta. The blood was then centrifuged (1,500 rpm/min) for 15 min to collect the serum, which was stored at −80 °C for subsequent analysis of fasting blood glucose, fasting insulin, follicle-stimulating hormone (FSH), luteinizing hormone (LH), and testosterone. The ovaries and uterus were removed, and the left ovary and a portion of the uterus were fixed with 4% paraformaldehyde, followed by dehydration, embedding, and sectioning for subsequent HE staining and immunohistochemistry. A portion of the uterus was fixed with electron microscopy fluid (G1102, Saiwei'er) for scanning electron microscopy experiments. The remaining fresh ovaries and uterus were stored at −80 °C for gene and protein expression analysis. After completing the experiments, rats were euthanized by cervical dislocation following re-anesthesia using the same method.

### 2.4 Knocking down endometrial *Hoxa11*

Rat *Hoxa11* RNA interference fragments (si-Hoxa11) were synthesized by GenePharma (Shanghai Jema Company, China), with the sense strand sequence GCCGUCUCGUCCAAUUUCUTT and the antisense strand sequence AGAAAUUGGACGAGACGGCTT. Fifteen PCOS model rats were selected, anesthetized, and a mixture of animal transfection reagent (Beijing InGen Biotechnology Co., Ltd.) and siRNA was injected into the uterine cavity. Among them, five rats were injected with si-NC on both sides of the uterine cavity, and the other ten were injected with si-Hoxa11 on both sides. They were then divided into three groups: si-NC control (PCOS + si-NC) group: intervened with physiological saline; si-Hoxa11 interference (PCOS + si-Hoxa11) group: intervened with physiological saline; si-Hoxa11 interference followed by QGW intervention (PCOS + si-Hoxa11 + QGW) group: intervened with QGW. Each group received equal volumes, once daily for 7 consecutive days. The rats were euthanized after being anesthetized with an intraperitoneal injection of 3% pentobarbital sodium, and the ovaries and uterus were removed and stored at −80 °C for gene and protein expression analysis.

### 2.5 Estrous cycle

The stages of the rat estrous cycle were determined based on vaginal cytology. Vaginal smears were collected between 9 and 10 a.m. each day. Vaginal epithelial cells were collected by flushing with physiological saline, fixed on slides, and stained with HE. The stages of the rat estrous cycle are divided into: Proestrus: mainly nucleated epithelial cells; Estrus: almost all anucleated cornified cells, which may be clustered; Metestrus: a large number of leukocytes and a few anucleated cornified cells; Diestrus: leukocytes, nucleated cells, and anucleated cornified cells coexist, with leukocytes predominating.

### 2.6 Hematoxylin and eosin (HE) staining

The ovarian tissue was fixed with 4% paraformaldehyde for 24 h, dehydrated with different concentrations of alcohol, embedded in paraffin, and sectioned. The largest cross-section of the ovary was taken and stained with HE to count the number of cystic follicles and the number of granulosa cell layers.

### 2.7 Enzyme-linked immunosorbent assay (ELISA)

ELISA kits (Jiangsu Enzyme Mark Biotechnology Co., Ltd.) were used to detect fasting blood glucose fasting insulin, FSH, LH, and testosterone levels in the serum of rats in each group. All steps of the ELISA kit were strictly followed according to the instructions, and the absorbance was measured at a wavelength of 450 nanometers using an enzyme-linked immunoassay reader (Thermo, China) to calculate the serum hormone content.

### 2.8 Real-Time fluorescence quantitative PCR

Total RNA from the uterus was extracted using the Trizol method. A cDNA synthesis chain kit (Hunan Aikorui Bioengineering Co., Ltd.) was used to remove DNA from RNA and reverse transcribe RNA into cDNA. A qRT-PCR kit (Hunan Aikorui Bioengineering Co., Ltd.) was employed for real-time fluorescence quantitative PCR (qRT-PCR). The qRT-PCR results were calculated using the *ΔΔ*Ct method, and the expression of target gene mRNA was expressed using the 2^-*ΔΔ*Ct method. Gapdh was used as the reference standard, and the relevant primers are listed in Table 1.

**Table 1:** Primer sequence.

Gene	Forward sequences	Reverse sequence
*Gapdh*	GGCACAGTCAAGGCTGAGAATG	ATGGTGGTGAAGACGCCAGTA
*Hoxa10*	AGTGCTGGGCTGTGTTTAATCA	CACGCTTCCATACTCTGCTCTT
*Hoxa11*	CCAATGACATACTCCTACTCCT	GGCTCAATGGCGTATTCTCT
*Glut4*	TTCCTCGCAGCACTTTAGCC	CACAGCCTAGCCACAACACA
*Igf1*	GCTCTTCAGTTCGTGTGTGG	TCCGAATGCTGGAGCCATAG
*Igfbp1*	ACTTCCGCTACTATCTACTCAG	GCCAAGAAACAACAGTTAGGA
*Il6*	AGCCAGAGTCATTCAGAGCA	TGGTCTTGGTCCTTAGCCAC
*Itgβ3*	TAGAAGAGCCTGAGTGTCCTAAG	TTCCAGATGAGCAGAGTAGCA
*Lif*	ATCAAGAGTCAACTGGCTCAACTCA	TGTTGGGCGCACATAGCTTATC

### 2.9 Immunoblotting

Total proteins were extracted from uterine tissue using RIPA lysis buffer (G2002-100ML, Servicebio) and phosphatase inhibitor (G2007-1ML, Servicebio). Sodium dodecyl sulfate-polyacrylamide gel electrophoresis (SDS-PAGE) was performed with varying concentrations based on the molecular weight of the proteins. Proteins were separated by electrophoresis and transferred onto polyvinylidene fluoride (PVDF) membranes (G6015-0.45, Servicebio). The PVDF membranes were immersed in a primary antibody solution and incubated overnight at 4 °C, with primary antibodies including *β-actin* (1:1250, AC026, ABclonal), *Hoxa10* (1:1250, A8550, ABclonal), *Hoxa11* (1:1250, A2976, ABclonal), *Glut4* (1:1250, A7637, ABclonal), *Igf1* (1:1250, A24744, ABclonal), Insulin-like growth factor binding protein-1(*Igfbp1*)(1:1250, A11109, ABclonal), *Il6* (1:1250, A21264, ABclonal), *Itgβ3* (1:1250, A19073, ABclonal), *Lif* (1:1250, A1288, ABclonal). After incubation with the secondary antibody (1:4000, AS014, ABclonal) at room temperature, signals were detected on a ChemiScope5300 (Shanghai Qinxiang Science) using ECL reagent (G2161-200ML, Servicebio), and the grayscale analysis was performed using Image J.

### 2.10 Immunohistochemistry

The uterus was fixed with 4% paraformaldehyde, dehydrated, embedded, and sectioned. Sections were deparaffinized with xylene and soaked in alcohol of varying concentrations. Antigen retrieval was performed using citrate retrieval solution (pH 6.0). Sections were soaked in a 3% hydrogen peroxide solution for 20 min at room temperature to block endogenous peroxidase activity, and then covered and sealed with 10% goat serum for 30 min. Primary antibodies included progesterone receptor(PR) (25871-1-AP, PTG) and estrogen receptor(ER) (18309-1-AP, PTG). Sections were incubated with the antibodies overnight at 4 °C, followed by incubation with the secondary antibody (PN0046, PinoFly Bio) for 1 h at room temperature. The sections were developed with DAB, counterstained with hematoxylin, dehydrated, and mounted. Under a microscope, six random fields were selected from each section, and the average optical density was calculated using Image.

### 2.11 Scanning electron microscopy

The uterus was fixed with electron microscopy fluid at room temperature for 2 h and then transferred to 4 °C for storage. After fixation, it was post-fixed with 0.1M phosphate buffer PB (pH 7.4, G0002, Saiwei'er) and osmium tetroxide (18456, Ted Pella Inc). It was dehydrated with ethanol of varying concentrations, dried using a critical point dryer (K850, Quorum), and then subjected to sample conduction treatment with an ion sputter coater (MC1000, HITACHI). The samples were observed and imaged under a scanning electron microscope (SU8100, HITACHI). Three random fields of view (10.0 μm) were selected for each sample, and then mature pinocytic protrusions were manually counted using Image J software under blind conditions.

### 2.12 Network pharmacology

Retrieve drugs from the TCMSP database (http://tcmspw.com/tcmsp.php), including *Pinellia ternata* (Thunb.), *Atractylodes lancea* (Thunb.), *Cyperus rotundus*, *Poria cocos*, *Tangerine peel*, *Ligusticum chuanxiong Hort*, and *Massa Medicata Fermentata*. The screening criteria for effective components of traditional Chinese medicine are oral bioavailability (OB) ≥30% and drug-likeness (DL) ≥0.18. The active components of *Massa Medicata Fermentata* are obtained through reference literature with PMIDs: 38299285 and 36527339. Potential target genes of the drugs are obtained from the TCMSP, DrugBank, and Swiss TargetPrediction databases (http://www.swisstargetprediction.ch/). Using |log2FC| ≥ 1 and corrected *p* < 0.05 as thresholds, download the GSE137354 dataset from the GEO database (platform GPL17586), which includes gene expression profiles of endometrium from 3 PCOS patients and 3 normal controls. Perform differential gene analysis of GSE137354 using R language limma with ggplot2 (3.3.6) and plot a volcano plot of differential genes (17). Convert drug target proteins to target gene IDs to obtain target genes, denoted as Qi Gong; then, denote the differential genes (DEGs) of the GSE137354 dataset as PCOS with Endometrial Disorder (PCOS-EM), and create a Venn diagram of the intersection to identify common targets. Import the intersecting target genes into the STRING database (18), select the species as “Homo sapiens,” and perform PPI analysis on the intersecting target genes. Further use the Degree algorithm in the cytoHubba plugin of Cytoscape to screen the intersecting target genes to obtain Hub genes. Search for corresponding active ingredients and drugs for the genes. To explore the biological pathways in which hub genes are involved in the disease, use the R package clusterprofile version 3.18.1 to perform single-gene pathway enrichment analysis on key genes, with a threshold of *p*-value < 0.05 and FDR < 0.25 as the criteria for selecting enriched pathways (19).

### 2.13 Molecular docking

Download the 3D structure of gene proteins from UniProt (https://www.uniprot.org/) and the 3D structure of active ingredients from PubChem (https://pubchem.ncbi.nlm.nih.gov/). Next, preprocess the protein receptor molecules: use AutoDock to remove water (20), ligands, cofactors, and ions that are not needed for docking from the predicted protein structures, add hydrogen, and finally obtain the 3D structure of the protein for subsequent molecular docking. Preprocess the ligand small molecules: first, convert the downloaded 2D structure SDF files to pdb format using Open Babel (21), then use the pybel module to convert the 2D structure of the small molecules to 3D structure; next, use AutoDock to add hydrogen and set it as a ligand. First, perform blind docking using AutoDock Vina (reason: blind docking is performed when there is no information about the catalytic site/binding site in the protein;), where the preprocessed 3D structures of the ligand and receptor molecules and the configuration file for all docking parameters are input. Finally, use PyMOL to visualize the results with the lowest binding energy (22).

## 3 Statistical analysis

Statistical analysis was performed using SPSS 24.0 software and GraphPad Prism 8 software. Data are expressed as the mean ± SEM. Inter-group comparisons were made using one-way analysis of variance (ANOVA). A *P*-value of less than 0.05 was defined as statistically significant.

## 4 Results

### 4.1 Network pharmacology analysis of QGW corresponding to endometrial receptivity genes in PCOS patients

**Figure 1:**
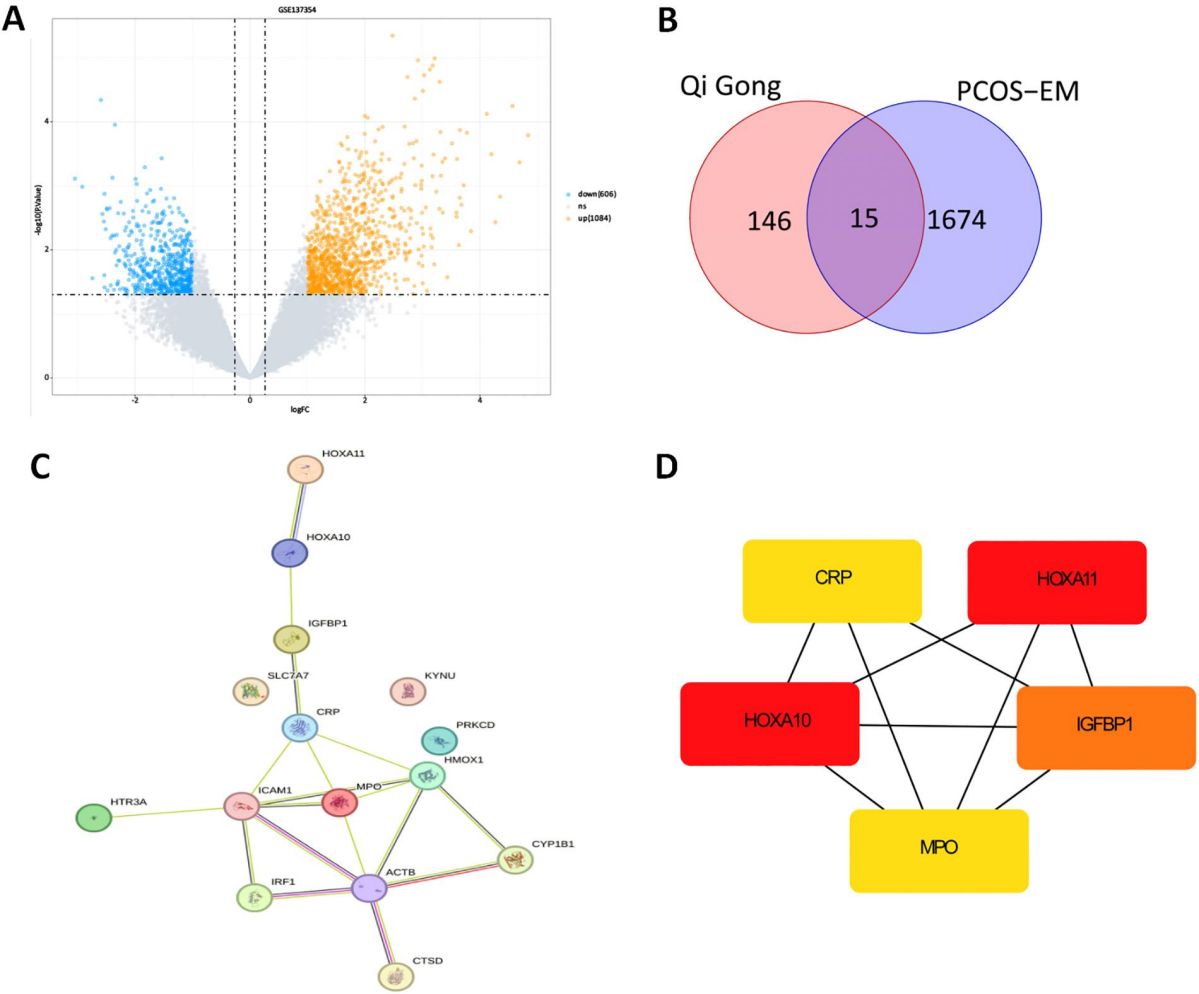
Network pharmacology analysis. **(A)** Volcano Plot of DEGs in GSE137354. **(B)** Venn Diagram of Potential Therapeutic Target Genes for QGW Treatment in PCOS-EM. **(C)** PPI Network Diagram of Targets of QGW in PCOS-EM. **(D)** Interaction Network Diagram of Hub Genes.

To ascertain the impact of Qigong Pill on the endometrial receptivity in PCOS, a network pharmacology analysis was conducted. Based on databases such as TCMSP/DrugBank and Swiss TargetPrediction, a total of 103 active components and 1527 protein targets were retrieved for QGW (Supplementary Table S1). Based on the GSE137354 dataset, 1,690 DEGs were identified in PCOS patients, of which 1,084 were upregulated and 606 were downregulated, as shown in the volcano plot (Figure 1A). The intersection of 161 target genes corresponding to the drug and 1,690 disease genes resulted in 15 potential therapeutic targets for drug-disease (Figure 1B). A PPI analysis was performed on the intersecting target genes, and a PPI network diagram of the 15 intersecting genes was constructed (Figure 1C). After screening the intersecting target genes and applying the Degree algorithm, five Hub genes were ultimately obtained: HOXA10, HOXA11, IGFBP1, CRP, and MPO (Figure 1D). Single-gene GO analysis indicated that HOXA10 and HOXA11 are involved in uterine development and embryonic development, while IGFBP1 is involved in the insulin receptor signaling pathway and positive regulation of cell growth. CRP is associated with innate immunity, inflammatory response, and acute inflammatory response. MPO is related to mechanical injury, fungal defense, and oxidative stress (Supplementary Figure S1).

### 4.2 Establishment of PCOS-IR rat model

**Figure 2:**
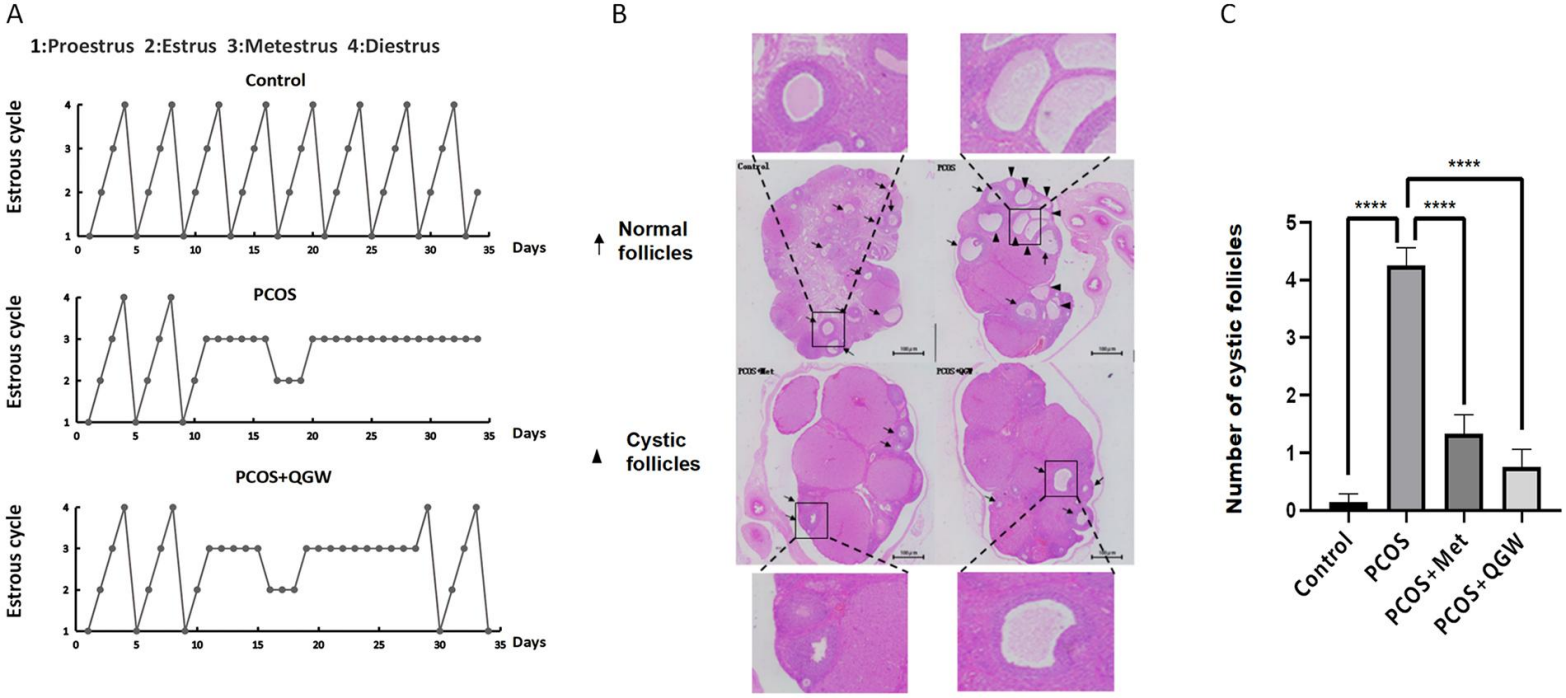
Estrous cycle, cystic follicles, and Granulosa cells in rats of each group. **(A)** Estrous Cycle in Rats of Each Group. **(B)** The number of cystic follicles and the layer number of granulosa cells, scale bar: 100 μm. **(C)** changes in the number of cystic follicles in rats after intervention. ****P < 0.0001.

The estrous cycle in the Control group showed regular changes, with one estrous cycle every 4 days, while the PCOS group had no cyclic changes and was arrested in the metestrus phase (Figure 2A). Serum testosterone, fasting insulin, and Homeostasis Model Assessment of Insulin Resistance (HOMA-IR) in the PCOS group were all increased compared to the Control group, with *P* < 0.01, which was statistically significant (Table 2). The number of cystic follicles in the PCOS group was increased compared to the Control group, and the granulosa cell layer was reduced, as seen in Figures 2B,C.

**Table 2:** Results of serum glucose and hormone levels in each group.

Indicator	Control (*n* = 5)	PCOS (*n* = 5)	PCOS + QGW (*n* = 5)	PCOS + Met (*n* = 5)	*P value*
Fasting blood-glucose (mmol/L)	38.85 ± 1.20	50.02 ± 4.14	39.06 ± 1.16	34.78 ± 4.31	0.0638^a^, 0.0382^b^, 0.0114^c^
Fasting insulin (mU/L)	80.00 ± 5.83	187.30 ± 19.84	120.70 ± 5.62	115.70 ± 6.47	<0.0001^a^, 0.0019^b^, 0.0045^c^
HOMA-IR	136.90 ± 6.86	400.80 ± 31.57	206.20 ± 13.17	180.90 ± 27.92	<0.0001^a^, <0.0001^b^, <0.0001^c^
Serum FSH level (IU/L)	12.18 ± 1.16	13.86 ± 1.54	9.086 ± 0.91	6.21 ± 1.00	0.7705^a^, 0.0274^b^, 0.0019^c^
Serum LH level (mIU/ml)	38.56 ± 2.24	53.22 ± 8.04	30.04 ± 2.95	32.91 ± 5.13	0.2633^a^, 0.0187^b^, 0.0920^c^
LH/FSH	3.29 ± 0.30	3.744 ± 0.21	3.35 ± 0.48	5.66 ± 0.83	0.8989^a^, 0.9113^b^, 0.0494^c^
Serum T level (pg/ml)	22.15 ± 2.20	37.40 ± 2.37	14.56 ± 1.76	26.72 ± 1.94	0.0002^a^, <0.0001^b^, 0.0137^c^

a Control vs. PCOS.

b PCOS vs. PCOS + QGW.

c PCOS vs. PCOS + Met.

### 4.3 QGW improves estrous cycle, sex hormone levels, and follicle quality in PCOS rats

After treatment with QGW (PCOS + QGW group), the estrous cycle in PCOS rats became more regular compared to the PCOS group (Figure 2A), and LH and serum testosterone (T) were significantly reduced, both with statistical significance (*P* < 0.01) (Table 2). After treatment with QGW, the number of cystic follicles in the ovaries of PCOS rats decreased (*P* < 0.01), and the number of granulosa cell layers increased (Figures 2B,C). PCOS model rats treated with metformin (PCOS + Met group) showed a decrease in LH/FSH and testosterone levels compared to the PCOS group, both with statistical significance (*P* < 0.01). In addition, both QGW and metformin were able to reduce the FSH level in PCOS rats (Table 2). Metformin was also able to reduce the number of cystic follicles and increase the number of granulosa cell layers in PCOS model rats (*P* < 0.01) (Figures 2B,C).

### 4.4 QGW reduces fasting blood glucose and insulin levels in PCOS-IR rats

After treatment with QGW (PCOS + QGW group), there was a significant reduction in fasting insulin, fasting blood glucose, and HOMA-IR in PCOS rats compared to the model group (PCOS group), with *P* < 0.01 indicating statistical significance. QGW demonstrated a similar effect to metformin, both being able to reduce fasting insulin, fasting blood glucose, and HOMA-IR in the PCOS model mice (Table 2). However, the reduction in fasting blood glucose levels by QGW was close to that of the control group, whereas metformin reduced blood glucose levels below those of the normal control group.

### 4.5 QGW increases the expression of endometrial receptivity markers and the number of pinopodes

**Figure 3:**
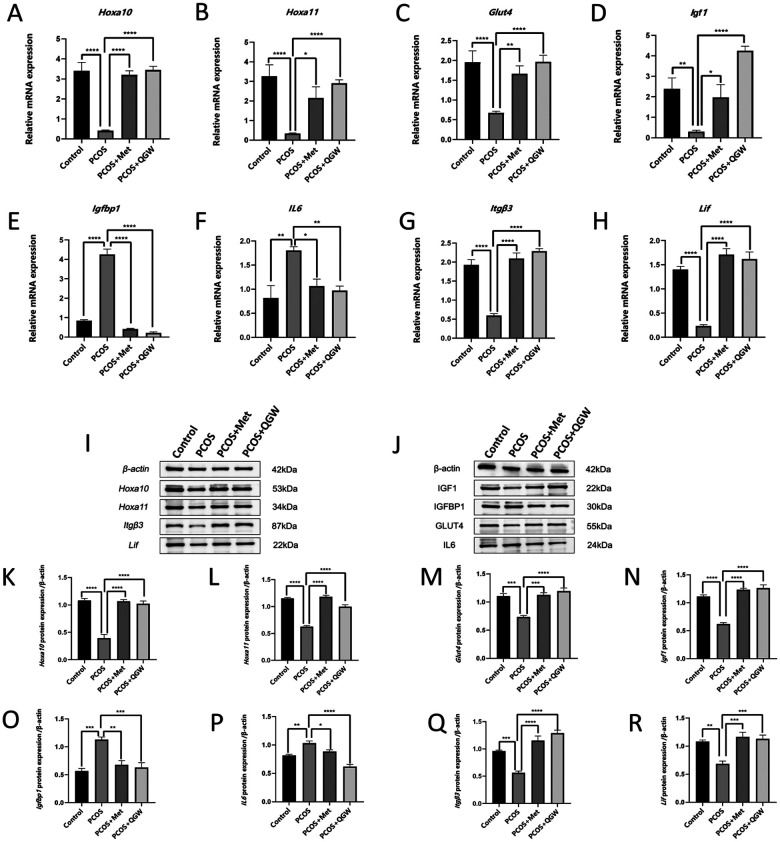
qRT-PCR and immunoblotting analyses of endometrial receptivity marker expression in each group of rats. (1) **(A–H)** RNA expression levels. (2) **(A–J)** Protein expression levels. *P < 0.05, **P < 0.01, ***P < 0.001, ****P < 0.0001, ns *P* > 0.05.

**Figure 4:**
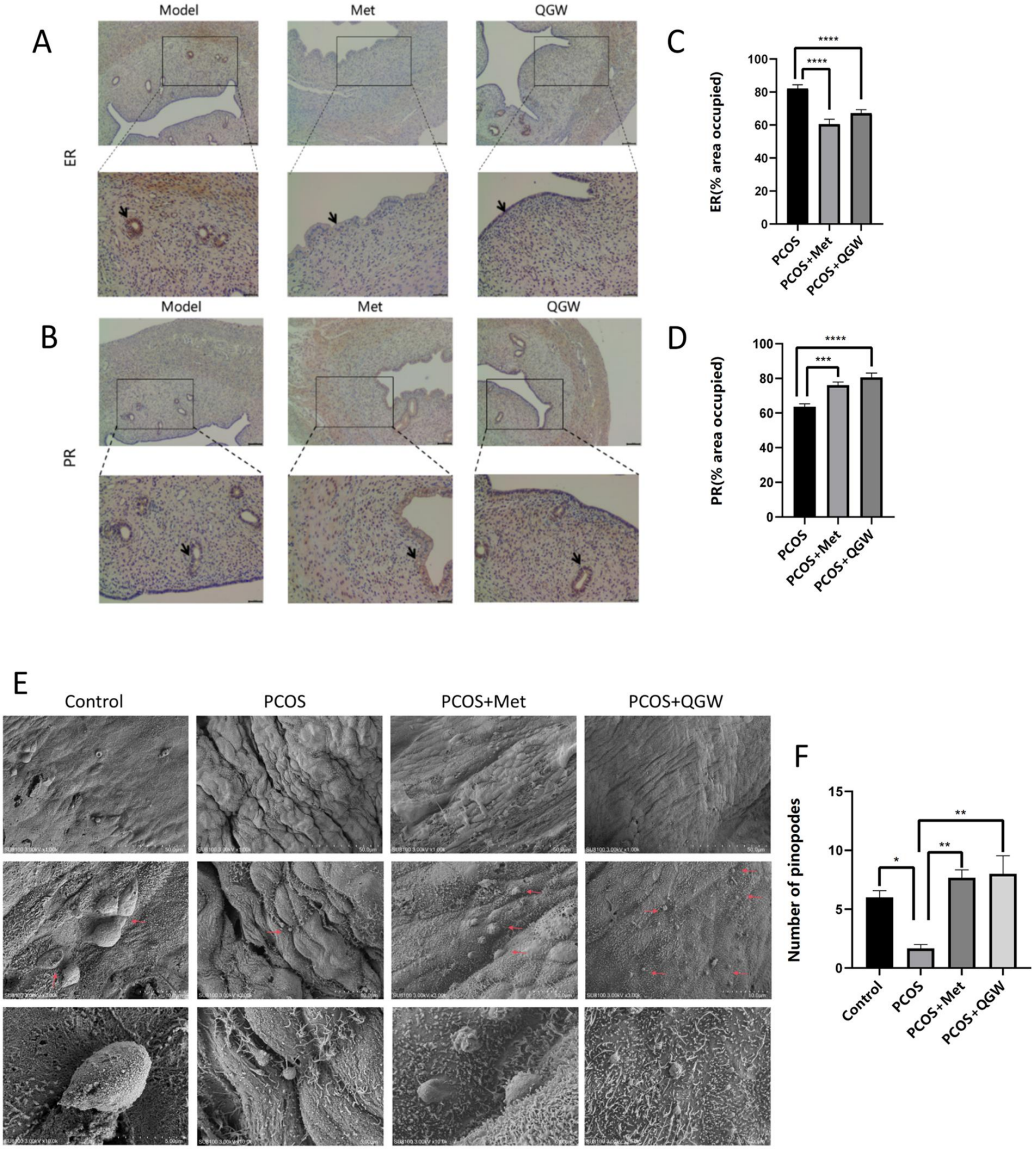
Detection of the expression of ER, PR, and pinopodes in the endometrium of PCOS rats after treatment. (1) Immunohistochemical staining for ER **(A–C)** and PR **(B–D)**. Positive cytoplasmic staining for ER and PR was detected in both epithelial and stromal cells. Magnification: × 400. Scale bar: 10 μm. ***P < 0.001, ****P < 0.0001. (2) The number of pinopodes in rats of each group.Red arrows indicate pinopodes. Scale bars: A, 50.0 μm; B, 10.0 μm. *P < 0.05, **P < 0.01.

Comparison of endometrial receptivity markers between the PCOS group and the control group revealed that the expression levels of *Hoxa10, Hoxa11, Glut4, Igf1, Itgβ3,* and *Lif* were decreased, while the expression levels of *Il-6* and *Igfbp1* were increased as shown by qRT-PCR and immunoblotting results (Figures 3A–R). The number of pinopodes in the endometrium of rats in the PCOS group was significantly reduced compared to the control group (Figures 4E,F). After treatment with QGW (PCOS + QGW group) or metformin (PCOS + Met group), compared to the PCOS group, qRT-PCR and immunoblotting results showed increased expression of *Hoxa10, Hoxa11, Glut4, Igf1, Itgβ3,* and *Lif,* and decreased expression levels of *Il-6* and *Igfbp1*.However, QGW showed a more pronounced effect in reducing the expression levels of *Il-6* (Figures 3A–R). Immunohistochemical results revealed an increase in PR expression and a decrease in ER expression in the PCOS + QGW group compared to the control group. Metformin treatment also showed similar therapeutic effects (Figures 4A–D). Scanning electron microscopy results indicated that, compared to the PCOS group, both metformin and QGW treatments increased the number of pinopodes in the endometrium of PCOS model rats (Figures 4E,F).

### 4.6 QGW improves the expression of endometrial receptivity markers through the Hoxa11-Itg*β*3 signaling pathway

**Figure 5:**
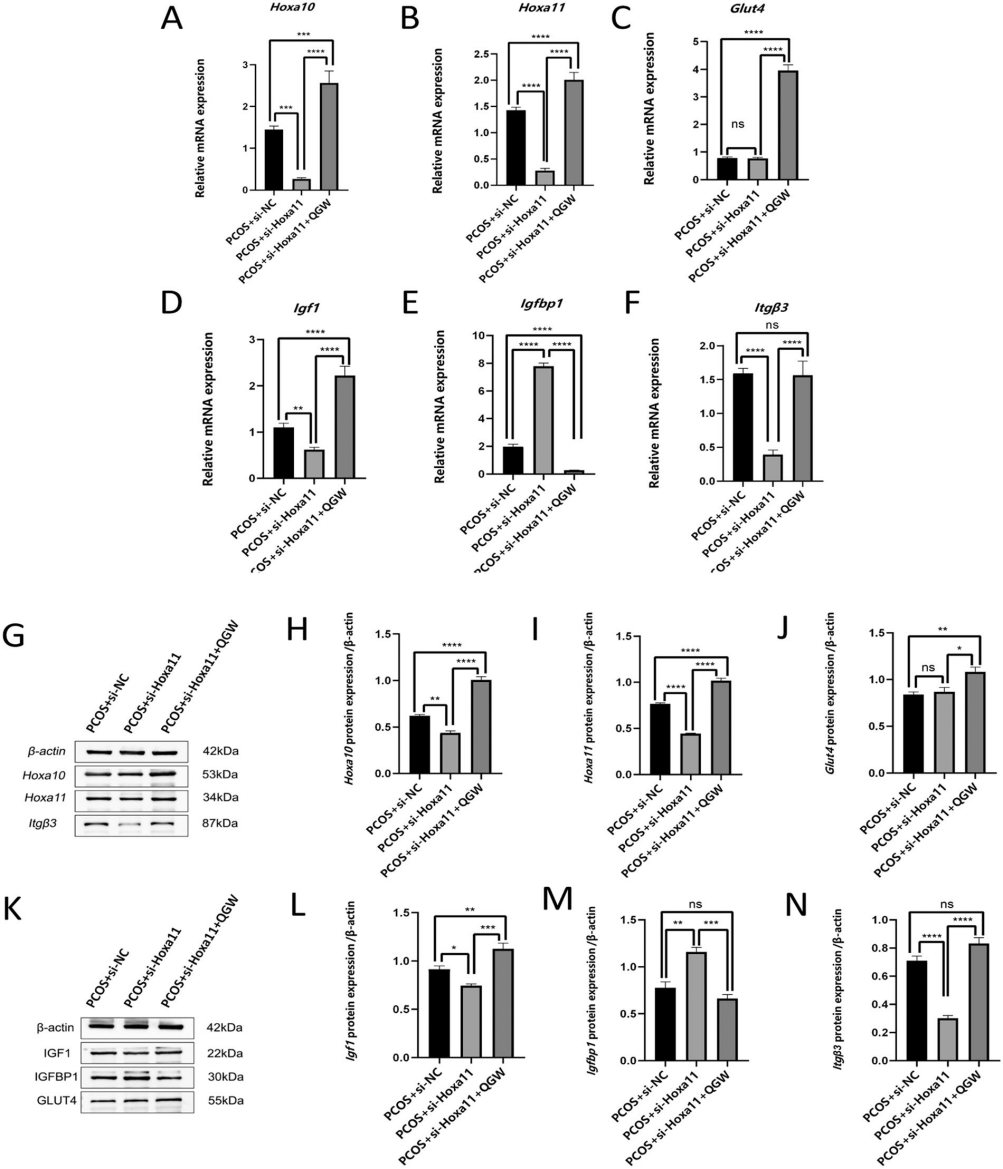
The effect of QGW on RNA and protein expression of endometrial receptivity genes in PCOS model rats, after knocking down *Hoxa11*. (1) **(A–H)** RNA expression levels. (2) **(A–J)** Protein expression levels. *P < 0.05, **P < 0.01, ***P < 0.001, ****P < 0.0001, ns *P* > 0.05.

To ascertain if the therapeutic effects of QGW are facilitated through the *Hoxa11-Itgβ3* signaling pathway in PCOS rats, we utilized si-Hoxa11 to selectively knock down *Hoxa11* in the endometrium of the PCOS model rats, creating the PCOS + si-Hoxa11 group for subsequent comparative analysis. Compared to the PCOS + si-NC group, qRT-PCR and immunoblotting analysis results showed that the RNA and protein expression levels of *Hoxa11* in the PCOS + si-Hoxa11 group were both reduced, indicating successful knockdown of the *Hoxa11* gene [Figures 5(1)B, G]. Compared to the PCOS + si-NC group, after knocking down *Hoxa11* (PCOS + si-HOXA11 group), the RNA and protein expression levels of *Hoxa10*, *Itgβ3*, and *Igf1* were significantly reduced, while the RNA and protein expression levels of *Igfbp1* were significantly increased, with *P* < 0.01 indicating statistical significance. There were no significant changes in the RNA and protein expression of *Glut4* in both groups, with *P* > 0.05, indicating no statistical significance. Compared to the PCOS + si-Hoxa11 group, the PCOS + si-Hoxa11 + QGW group (after treatment with QGW) showed increased RNA and protein expression of *Hoxa10*, *Hoxa11*, *Itgβ3*, *Glut4*, *and Igf1*, and decreased RNA and protein expression levels of *Igfbp1*, with *P* < 0.001 indicating statistical significance. In contrast to the PCOS + si-NC group, the PCOS + si-Hoxa11 + QGW group exhibited enhanced RNA and protein expression for *Hoxa10*, *Hoxa11*, *Glut4*, and *Igf1*, with *P* < 0.001 signifying statistical significance (Figure 5). Meanwhile, the protein expression levels of *Igfbp1* and *Itgβ3* remained unaltered, with *P* > 0.05 suggesting no statistical significance (Figures 5G–N). When compared to the PCOS + si-NC group, the PCOS + si-Hoxa11 + QGW group displayed no significant alterations in the RNA expression of *Itgβ3* (*P* > 0.05), yet there was a marked decrease in the RNA expression of *Igfbp1*, with *P* < 0.001 denoting significant biological statistical significance (Figures 5A–F).

### 4.7 Molecular docking

**Figure 6:**
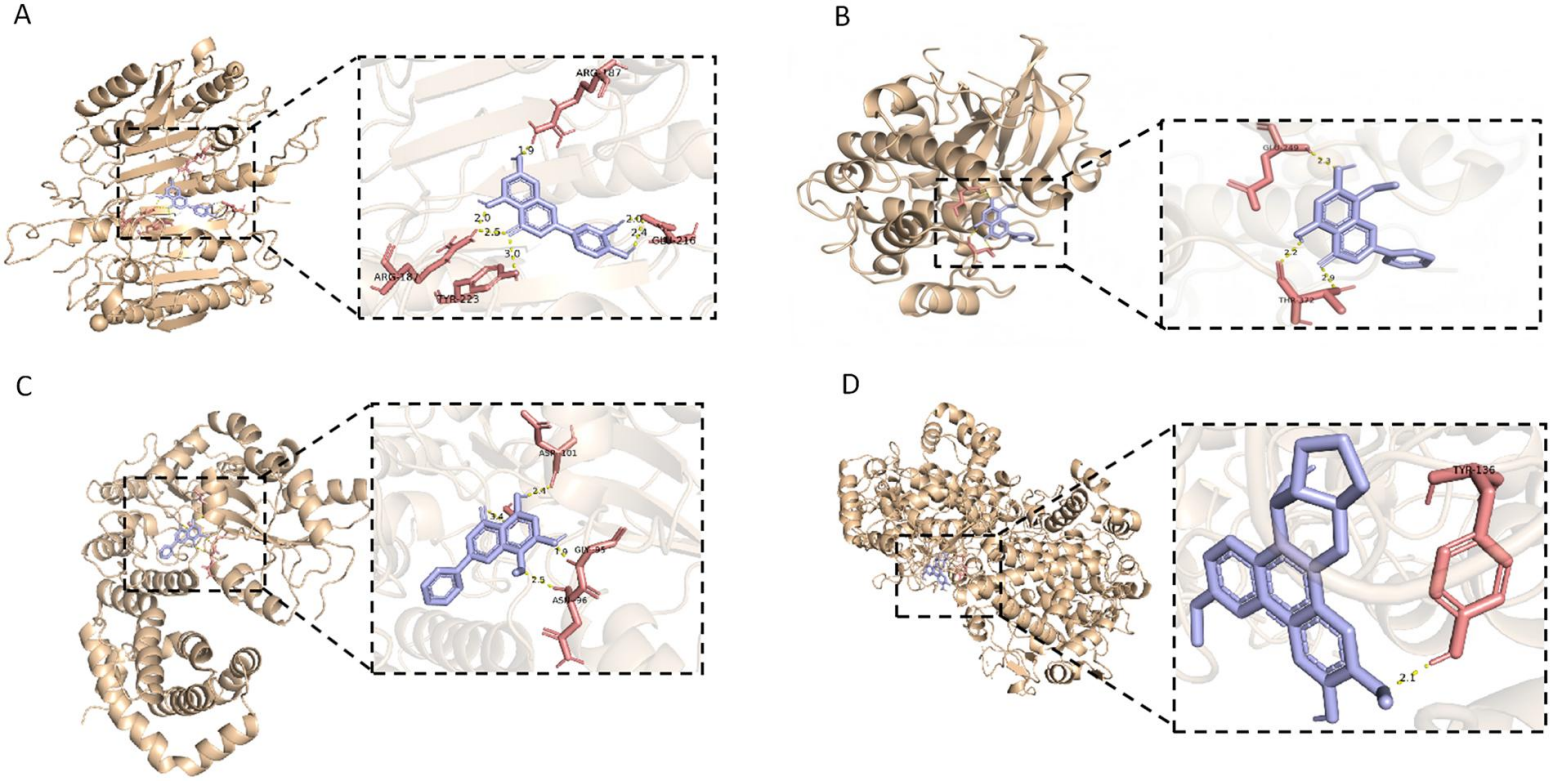
Molecular docking. **(A–C)** Show the docking of HOXA10, HOXA11, and IGFBP1 with wogonin, while **(D)** shows the docking of IL-6 with luteolin. Yellow bonds represent hydrogen bonds, red indicates the binding residues, and purple represents the 3D structure of the active compounds.

QGW has identified a total of 103 active components, among which wogonin, luteolin, lauric acid, methyl palmitate, quercetin, and gamma-aminobutyric acid were found to act on the genes HOXA10, HOXA11, IGFBP1, and IL-6 (Supplementary Table S2). HOXA10, HOXA11, and IGFBP1 were docked with wogonin, while IL-6 was docked with luteolin. The results of molecular docking are represented by the binding energy, which indicates the binding activity between the receptor and ligand. A binding energy of less than 0 KJ/mol indicates that the ligand and receptor have the ability to spontaneously bind. The smaller the binding energy, the higher the affinity between the receptor and ligand, and the greater the likelihood of interaction. A binding energy of less than −1.2 kcal/mol (−5 KJ/mol, 1 Kcal = 4.184 KJ) indicates high binding activity, while a binding energy of less than −1.7 Kcal/mol (−7.0 KJ/mol) indicates very strong binding activity. The molecular docking results (Table 3; Figure 6) show that there is a certain degree of interactive force between the targets and their corresponding active ingredients, forming multiple hydrogen bonds, which are important forces in the interaction. In summary, the docking binding free energy of less than −5 indicates that HOXA10, HOXA11, IGFBP1, and IL-6 have good binding capabilities with their corresponding active ingredients.

**Table 3:** Molecular docking results of Key genes.

Gene	Molecule name	Molecular docking binding energy (kcal/mol)
HOXA10	Wogonin	−7.4
HOXA11	Wogonin	−6.2
IGFBP1	Wogonin	−6.1
IL-6	Luteolin	−6

IL-6 has a molecular docking binding energy greater than 0 with other active components, hence no analysis docking is performed.

## 5 Discussion

Current studies have found that QGW can inhibit granulosa cell apoptosis, enhance the quality of oocytes (12, 13), and promote follicular development and ovulation (14), thereby increasing the pregnancy rate in PCOS patients. However, infertility in PCOS is not solely due to ovulatory disorders; abnormal endometrial receptivity is also one of the factors contributing to infertility in PCOS patients. It has been observed that after taking QGW, PCOS patients experience an increase in endometrial thickness, a decrease in the uterine artery RI, and an increase in pregnancy rates (11, 16), suggesting that QGW may improve endometrial receptivity in PCOS patients. Through network pharmacology analysis, the study found that QGW can act on HOXA10, HOXA11, IGFBP1, CRP, and MPO in the endometrium of PCOS patients. Single-gene GO analysis indicates that HOXA10 and HOXA11 are involved in uterine and embryonic development, while IGFBP1 is involved in the insulin receptor signaling pathway and positively regulates cell growth. CRP is associated with innate immunity, inflammatory response, and acute inflammatory response. MPO is related to mechanical injury, fungal defense, and oxidative stress. The reduced expression of HOXA10, HOXA11, LIF, ITGB3, and GLUT4 in the endometrium of PCOS patients is associated with decreased endometrial receptivity in PCOS and may lead to infertility related to PCOS (6–8). IGFBP1 is expressed by human decidualized endometrial mesenchymal cells and acts as a negative factor in embryo implantation, participating in the decidualization process (23).This study investigates the effect of QGW on endometrial receptivity in rats with IR-PCOS. Considering that there is only one dataset available for the endometrium of PCOS patients, there is a concern about data bias. In conjunction with the literature and our research objectives, this has led to the subsequent selection of endometrial receptivity genes such as HOXA10, HOXA11, ITGB3, LIF, GLUT4 and IGFBP1 for animal experimental research.

The establishment of the PCOS-IR rat model through subcutaneous injection of DHEA combined with a high-fat diet resulted in rats exhibiting arrested estrous cycles, increased fasting insulin, HOMA-IR, and testosterone levels, as well as an increase in cystic follicles and a decrease in the number of granulosa cell layers in ovarian tissue, indicating the successful construction of an PCOS-IR model. Metformin, an insulin sensitizer, has been widely confirmed for the treatment of PCOS patients. Metformin can improve blood lipid and glucose levels, testosterone, progesterone,LH, FSH, and estradiol levels in PCOS model rats, and can improve IR in PCOS rats through the PI3K-Akt-mTOR and AMPK signaling pathways (24), reducing the risk of late miscarriage and preterm birth (25). Therefore, this study uses metformin as a positive control to evaluate the therapeutic effect of QGW on PCOS-IR rat model.

Serum testosterone levels in PCOS patients are significantly elevated, and increased testosterone levels can alter the endometrial environment, making it unfavorable for embryo implantation and leading to recurrent miscarriage (26, 27). In PCOS patients, FSH levels remain unchanged or decrease, while LH levels increase, leading to an elevated LH/FSH ratio, which is an important diagnostic marker and therapeutic indicator for PCOS (28). This study found that after treatment with QGW (PCOS + QGW group) or metformin (PCOS + Met group), testosterone levels in PCOS model rats were lower than those treated with physiological saline (PCOS group). QGW significantly reduced LH levels, which was statistically significant, while metformin significantly reduced the LH/FSH ratio. Both drugs improved the endometrial environment by reducing hormone levels in the body, but through different mechanisms. PCOS patients, 50%–70% of whom have impaired glucose tolerance, hyperinsulinemia, and IR, have IR as an indirect factor leading to anovulation (29). IR and hyperinsulinemia in PCOS patients have a detrimental impact on endometrial function and receptivity (30). The results of this experimental study showed that after treatment with metformin (PCOS + Met group) or QGW (PCOS + QGW group), fasting blood glucose, fasting insulin, and HOMA-IR in model rats were all reduced, indicating that both QGW and metformin can improve IR and glucose metabolism abnormalities in PCOS, thereby improving endometrial receptivity. The above results suggest that both QGW and metformin can improve glucose metabolism and sex hormone levels in PCOS. However, metformin exhibited a more significant reduction in fasting blood glucose levels, even lower than those of the Control group, potentially explaining why some patients report dizziness and discomfort following metformin administration. Although QGW can reduce fasting blood glucose levels, it did not lower blood sugar below the levels of the Control group.

Endometrial receptivity-related molecular markers are crucial for normal implantation. In normal individuals, decidualized stromal cells typically express high levels of HOXA10, HOXA11, ITGB3, LIF, and prolactin (PRL), etc (31–33). IGFBP1 is expressed by human decidualized endometrial mesenchymal cells and acts as a negative factor in embryo implantation, participating in the decidualization process (23). ITGB3 has been widely recognized as an embryo attachment factor between the endometrium and the trophoblast (34). LIF, a pleiotropic cytokine, is essential for the progression of normal pregnancy (35). Downregulation of LIF protein expression disrupts the formation of phagocytic cups in the endometrial epithelial cells, impairs endometrial receptivity, and inhibits embryo implantation (36, 37). Progesterone and estrogen are two cholesterol-derived steroid hormones that synergistically mediate changes in the structure and function of the uterus, preparing it for blastocyst implantation (38). HOX genes encode transcription factors with homeodomains, which are dynamically expressed in the endometrium and are essential for the growth, differentiation, and implantation of the endometrium. In the human endometrium, the expression of HOXA10 and HOXA11 is regulated by progesterone, and they peak during implantation (39). Studies have shown that in patients with PCOS, the expression of HOXA10, HOXA11, LIF, ITGB3, and GLUT4 in the endometrium is reduced, which is associated with decreased endometrial receptivity in PCOS and may lead to PCOS-related infertility (6–8). The decidualization response of endometrial stromal cells in PCOS patients is impaired, with decreased PR expression and increased ER expression (32, 40). These findings indicate that the expression of endometrial markers in PCOS patients is abnormal, leading to compromised endometrial receptivity. In this experiment, compared with the Control group, the PCOS group showed reduced expression levels of *Hoxa10*, *Hoxa11*, *Glut4*, *Igf*, *Itgβ3*, *Lif*, and PR proteins in the uterus, and increased ER expression, indicating that our established PCOS rat model has impaired endometrial receptivity. After treatment with QGW in PCOS rats, the RNA and protein expression of *Hoxa10*, *Hoxa11*, *Glut4*, *Igf*, *Itgβ3*, and *Lif* in the uterus of the PCOS + QGW group were increased, the expression of ER protein was decreased, and the expression of PR protein was increased, indicating that QGW improves endometrial receptivity in PCOS rats by increasing the levels of endometrial receptivity markers. Additionally, the number of pinopodes is positively correlated with blastocyst implantation (41). PCOS patients have a reduced number of pinopodes and smaller volume (42). We observed changes in the number of endometrial pinopodes through electron microscopy and found that the number of pinopodes in the endometrium of PCOS rats treated with QGW significantly increased, further proving that QGW can improve endometrial receptivity. In the proliferative phase endometrium of PCOS patients, inflammatory factors such as IL6 are increased (43, 44). Our research results also confirmed that the expression of *Il6* in the endometrium of PCOS rats is elevated, and treatment with QGW can reduce the expression of *Il6* in the endometrium of PCOS rats, indicating that QGW can improve the inflammatory state of the PCOS endometrium, thereby improving endometrial receptivity.

Patients with PCOS exhibit alterations in genes related to insulin signaling in the local endometrium, which affects local glucose metabolism and endometrial receptivity (32, 45). Our study found that compared to the Control group, the protein expression levels of *Igfbp1* in the endometrium of the PCOS model group were increased, while the protein expressions of *Glut4* and *Igf1* were decreased, indicating that the DHEA-induced PCOS model rats showed changes in insulin signaling-related genes and glucose metabolism disorders in the local endometrium, which is different from the results in the peripheral blood of PCOS patients (46). After treatment with QGW, the protein expression of *Igfbp1* in the endometrium of PCOS model rats was reduced, and the protein levels of *Glut4* and Igf1 were increased, indicating that QGW can affect local insulin signaling and glucose metabolism in the endometrium. GLUT4 regulates glucose uptake in cells. IGF1, in conjunction with insulin, participates in the implantation process, and IGF1, in combination with insulin, is involved in the process of embryo implantation, and the IGF-binding capacity of IGFBP1 regulates the effects of IGFs within the endometrium (47), indirectly suggesting that QGW may influence the overall endometrial receptivity by affecting changes in local insulin signaling and glucose metabolism.

This study knocked down the expression of *Hoxa11* in the endometrium of a PCOS -IR rat model using small RNA interference fragments. After silencing *Hoxa11* expression, the expression of *Hoxa10*, *Igf1*, and *Itgβ3* was reduced, while the expression of *Igfbp1* was significantly increased, and the expression of *Glut4* remained unchanged. This suggests that *Hoxa11* may positively regulate the expression of *Hoxa10*, *Igf1*, and *Itgβ3*, negatively regulate the expression of *Igfbp1*, and not regulate the expression of *Glut4*. This is consistent with the mouse endometrium Hox genes, which negatively regulate the expression of *Igfbp1* and *Itgβ3 (*33). Compared with the PCOS + si-Hoxa11 group, the PCOS + si-Hoxa11 + QGW group showed increased expression of *Hoxa11*, *Hoxa10*, *Glut4*, and *Itgβ3*, and decreased expression of *Igfbp1*. Compared with the interference control group(the PCOS + si-NC group), the PCOS + si-Hoxa11 + QGW group showed significantly increased expression of *Hoxa11*, *Hoxa10*, *Igf1*, and *Glut4*, with no statistically significant changes in the expression of *Itgβ3* and *Igfbp1*. This indicates that QGW regulates the expression of *Itgβ3* and *Igfbp1* in the endometrium by increasing the expression of *Hoxa11*, and the regulation of *Hoxa10* and *Igf1* expression is partly achieved through *Hoxa11*. The effect of QGW on *Glut4* expression is independent of *Hoxa11* and may involve other mechanisms.

QGW has identified a total of 103 active components, among which wogonin, luteolin, lauric acid, methyl palmitate, quercetin, and gamma-aminobutyric acid were found to act on the genes HOXA10, HOXA11, IGFBP1, and IL-6. Molecular docking studies indicated that HOXA10, HOXA11, and IGFBP1 dock with wogonin, while IL-6 docks with luteolin. No active components were identified as being associated with ITGB3, IGF1, GLUT4, and LIF in the drug target analysis. This absence of association may be attributed to several factors: firstly, the active components of QGW may directly influence HOXA10, HOXA11, and IGFBP1 without directly impacting ITGB3, IGF1, GLUT4, and LIF, suggesting that QGW could indirectly modulate the expression of ITGB3, IGF1, and GLUT4 through the mediation of HOXA11 and IGFBP1. Secondly, it cannot be ruled out that there may be inherent biases in the network pharmacology analysis, and the limitations of the dataset must also be taken into account. Therefore, our future work plan is to conduct animal experiments to elucidate the effective active components that enhance the expression of endometrial receptivity genes in PCOS. It is worth noting that this study did not perform ChIP, EMSA, or promoter reporter assays. Therefore, it remains unclear whether Hoxa11 directly binds to the promoters of *Itgβ3* or *IGFBP1*. The proposed *Hoxa11-Itgβ3/IGFBP1* regulatory axis remains speculative and requires further experimental verification.

 QGW is a multi-component formula in which multiple active ingredients may act synergistically. The inferences regarding wogonin in this study are based solely on network pharmacology and molecular docking analyses, without in vivo or in vitro validation using the purified compound. Future work will include experiments such as single-compound intervention, formula disassembly, and component knockout/supplementation to elucidate the actual functional contribution of wogonin.

## 6 Conclusions

QGW is capable of enhancing the expression of genes related to endometrial receptivity in PCOS model rats and increasing the number of pinopodes, thereby improving the endometrial receptivity in PCOS rats. The results suggest that QGW may improve endometrial receptivity potentially through upregulation of *Hoxa11* accompanied by increased *Itgβ3* and decreased *Igfbp1*. However, whether *Hoxa11* directly binds to the promoter regions of relevant genes requires further validation. Network pharmacology and molecular docking indicate that wogonin may be a potential active component with considerable potential, possibly involved in regulating targets such as *HOXA10, HOXA11,* and *IGFBP1*, but its specific contribution still needs to be further verified through in vivo and in vitro functional experiments, so it is not clear whether wogonin is a necessary or key component.

## Data Availability

The original contributions presented in the study are included in the article/Supplementary Material, further inquiries can be directed to the corresponding author.
