# Correction: The role and underlying mechanisms of Qi Gong Wan in enhancing the endometrial receptivity of a rat model with polycystic ovary syndrome

**DOI:** 10.3389/frph.2026.1826232

**Published:** 2026-03-13

**Authors:** Frontiers Production Office

**Affiliations:** Frontiers Media SA, Lausanne, Switzerland

**Keywords:** endometrial receptivity, herb, infertility, insulin resistance, polycystic ovary syndrome, Qi Gong Wan

Authors Chun Ding and Qinhua Li were erroneously omitted as equal contributing authors.

The correct author list is:

Chun Ding^1,2,3†^, Qinhua Li^1*†^, Yiwen Deng^1,2†^, Yuhan Liu^1,2†^, Lei Liu^1†,^ ChunYu Cao^3^ Siwei Li^1,2^, Tingting Zheng^1^, Liangqun Xie^1^, Jing Zhao^1^, Hong Ye^1^ and Junkui Li^4^

The original version of this article has been updated.

