# Supplementary material for: The role and underlying mechanisms of Qi Gong Wan in enhancing the endometrial receptivity of a rat model with polycystic ovary syndrome
Source: Front Reprod Health. 2026 Mar 3;7:1733583. doi: 10.3389/frph.2025.1733583 (PMC13023406; doi:10.3389/frph.2025.1733583)
Supplement: Supplementary file 1 [file Table1.docx]

| Chinese Medicine Name | Number of Components | | | Number of Targets | | | |
| --- | --- | --- | --- | --- | --- | --- | --- |
|  | All | After Screening（OB30 DL0.18） | | All | | After Screening (Deduplicated) | |
| Pinellia ternata (Thunb.) | 116 | | 13 | | 1302 | | 386 |
| *Atractylodis Rhizoma* | 49 | | 9 | | 276 | | 93 |
| *Cyperi Rhizoma* | 104 | | 19 | | 1029 | | 275 |
| *Poria cocos* | 34 | | 15 | | 121 | | 69 |
| *Tangerine peel* | 63 | | 5 | | 479 | | 147 |
| *Ligusticum chuanxiong Hort* | 189 | | 7 | | 1356 | | 223 |
| *Massa Medicata Fermentata* | 55 | | 35 | | 686 | | 334 |
| Total | 610 | | 103 | | 5249 | | 1527 |

Supplementary Table1: Statistics of Drug Components and Target Genes
