# Supplementary material for: The role and underlying mechanisms of Qi Gong Wan in enhancing the endometrial receptivity of a rat model with polycystic ovary syndrome
Source: Front Reprod Health. 2026 Mar 3;7:1733583. doi: 10.3389/frph.2025.1733583 (PMC13023406; doi:10.3389/frph.2025.1733583)
Supplement: Supplementary file 2 [file Table2.docx]

| Gene | Active Component ID | Active Component | Source of Active Component |
| --- | --- | --- | --- |
| HOXA10 | MOL000173 | wogonin | *Atractylodis Rhizoma* |
| HOXA11 | MOL000173 | wogonin | *Atractylodis Rhizoma* |
| IGFBP1 | MOL000173 | wogonin | *Atractylodis Rhizoma* |
| IL-6 | MOL000006 | luteolin | *Cyperi Rhizoma* |
|  | MOL000006 | luteolin | *Massa Medicata Fermentata* |
|  | MOL000305 | lauric acid | *Poria cocos* |
|  | MOL000879 | Methyl palmitate | *Ligusticum chuanxiong Hort* |
|  | MOL000305 | lauric acid | *Tangerine peel* |
|  | MOL000173 | wogonin | *Atractylodis Rhizoma* |
|  | MOL000388 | gamma-aminobutyric acid | *Pinellia ternata (Thunb.)* |

Supplementary Table 2 .Active Components Corresponding to Key Genes
