# Supplementary material for: The role and underlying mechanisms of Qi Gong Wan in enhancing the endometrial receptivity of a rat model with polycystic ovary syndrome
Source: Front Reprod Health. 2026 Mar 3;7:1733583. doi: 10.3389/frph.2025.1733583 (PMC13023406; doi:10.3389/frph.2025.1733583)
Supplement: Supplementary file 3 [file Datasheet1.pdf]

HOXA10

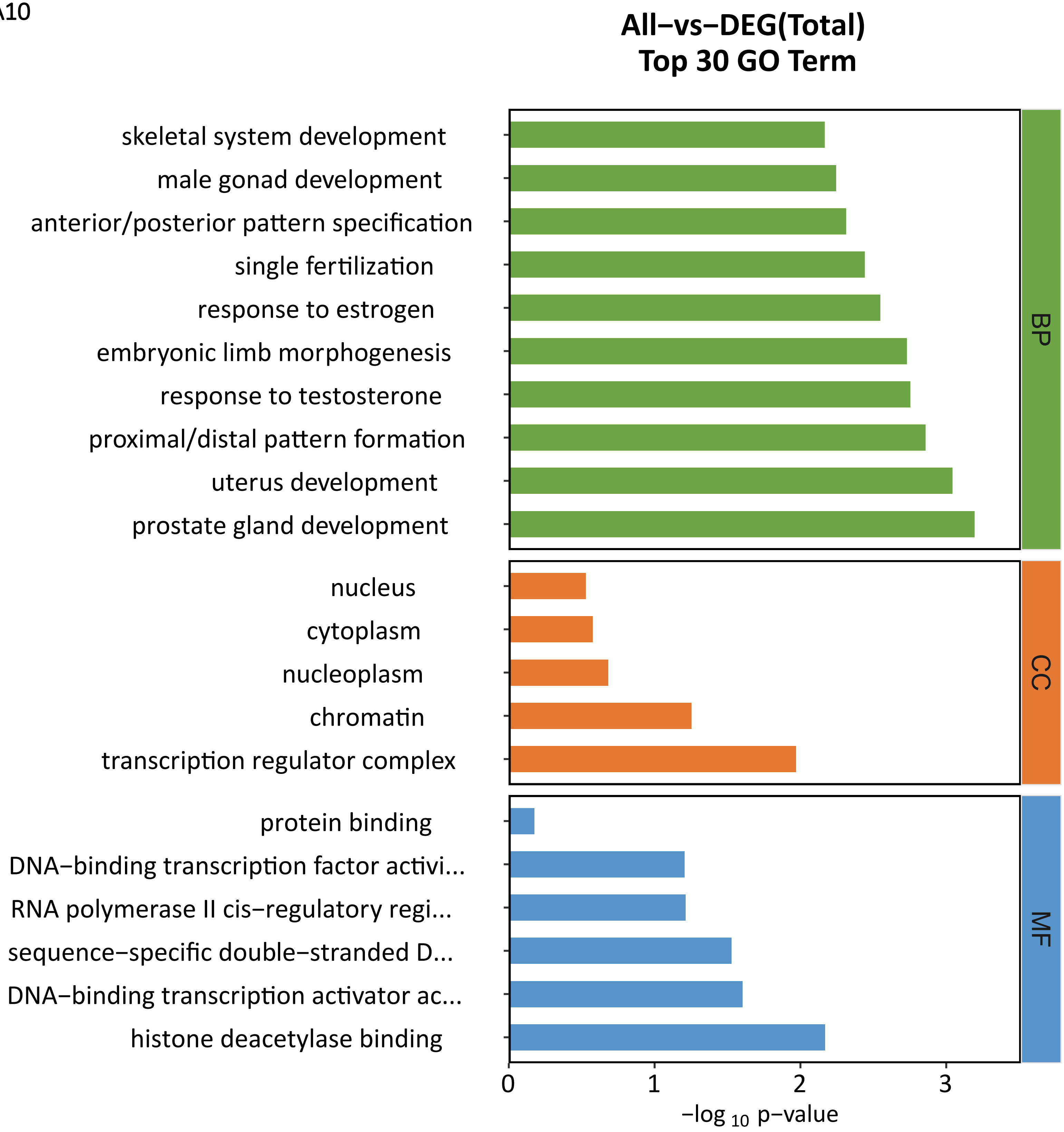
All−vs−DEG(Total)
Top 30 GO Term
skeletal system development
male gonad development
anterior/posterior pattern specification
single fertilization
response to estrogen
embryonic limb morphogenesis
response to testosterone
proximal/distal pattern formation
uterus development
prostate gland development
nucleus
cytoplasm
nucleoplasm
chromatin
transcription regulator complex
protein binding
DNA−binding transcription factor activi...
RNA polymerase II cis−regulatory regi...
sequence−specific double−stranded D...
DNA−binding transcription activator ac...
histone deacetylase binding
BP
CC
MF
0
1
2
3
−log 10 p−value

HOXA11

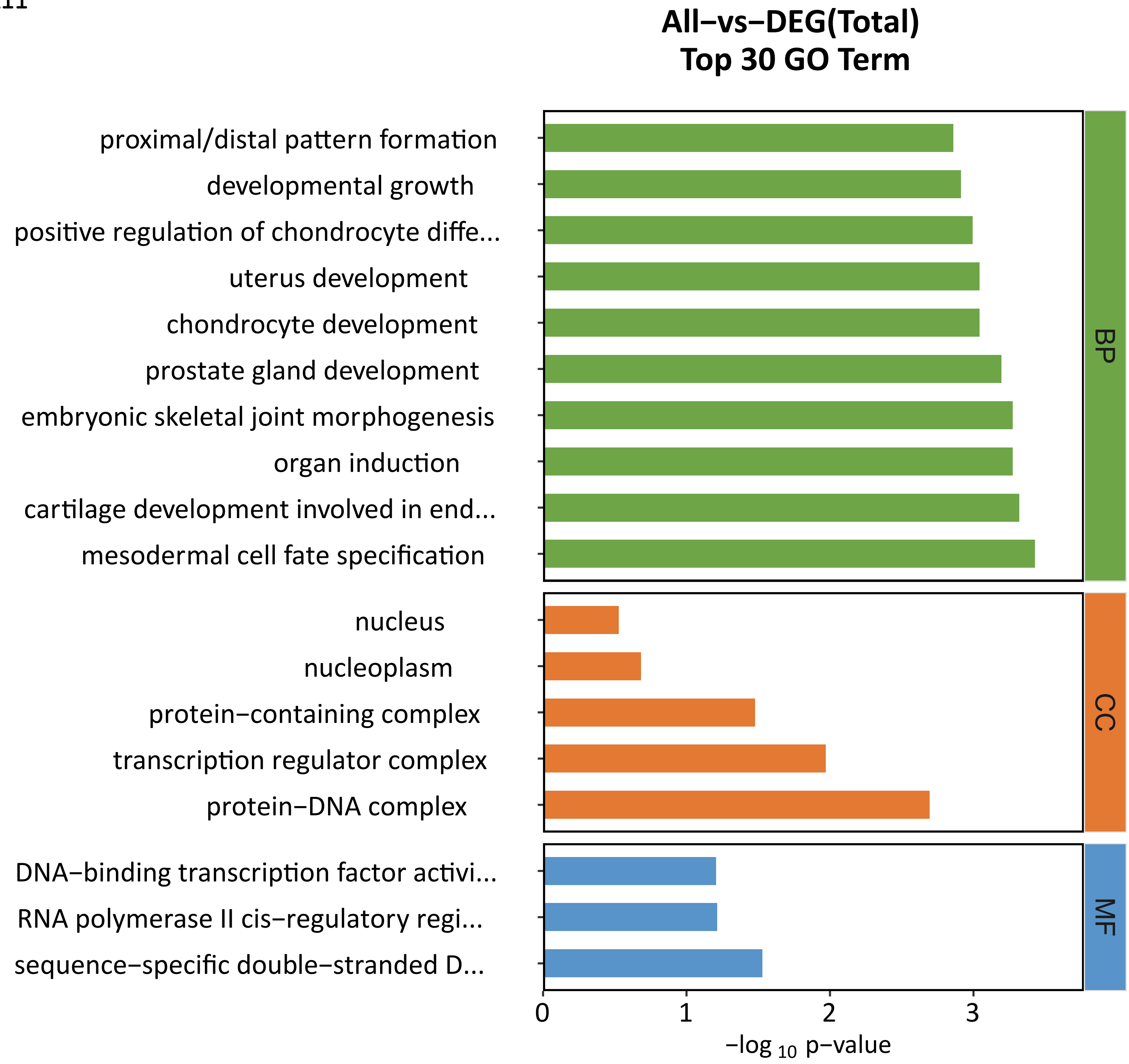

All−vs−DEG(Total)
Top 30 GO Term
proximal/distal pattern formation
developmental growth
positive regulation of chondrocyte diffe...
uterus development
chondrocyte development
prostate gland development
embryonic skeletal joint morphogenesis
organ induction
cartilage development involved in end...
mesodermal cell fate specification
nucleus
nucleoplasm
protein−containing complex
transcription regulator complex
protein−DNA complex
DNA−binding transcription factor activi...
RNA polymerase II cis−regulatory regi...
sequence−specific double−stranded D...
BP
CC
MF
0
1
2
3
−log 10 p−value

IGFBP1

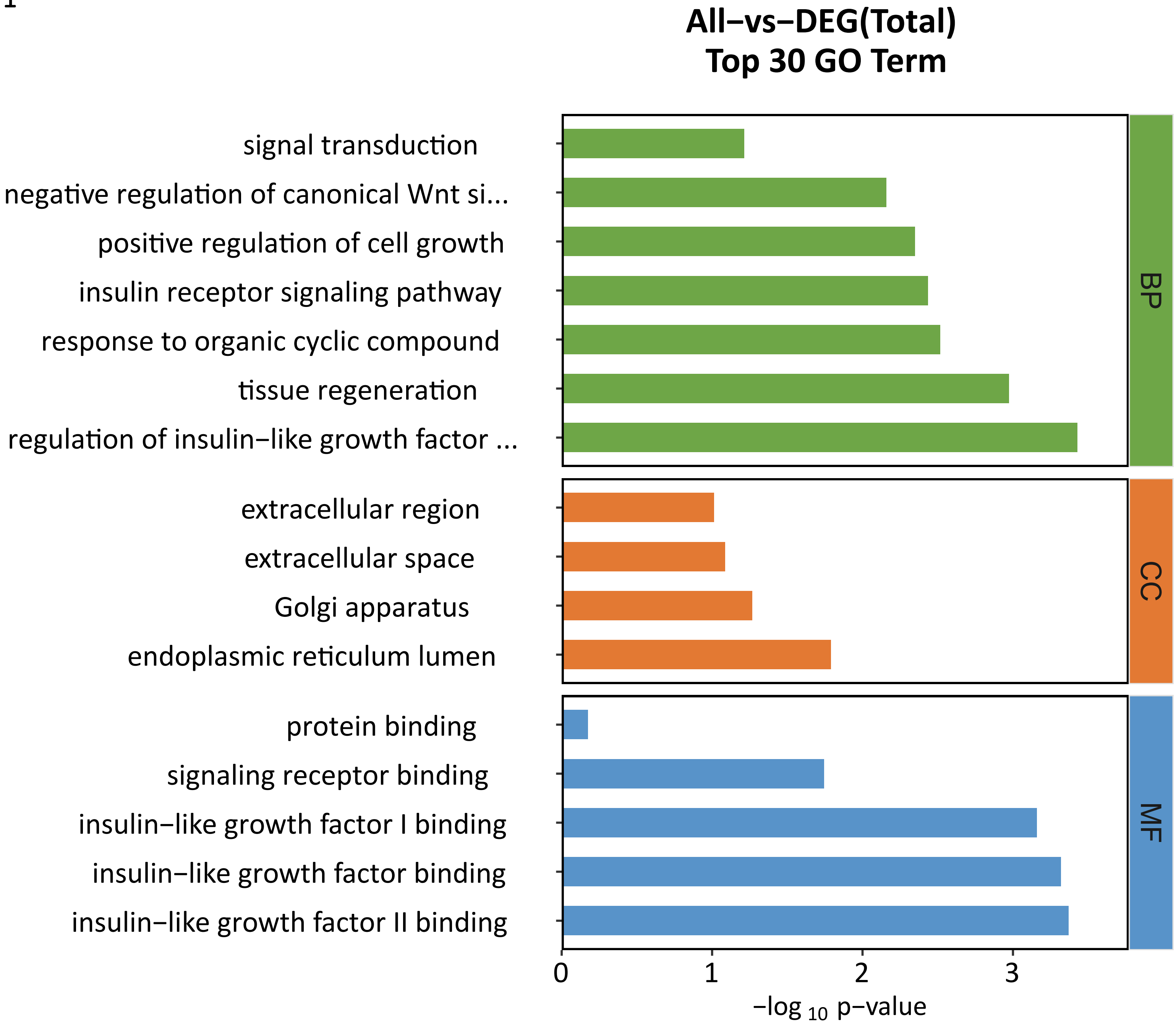

All−vs−DEG(Total)
Top 30 GO Term
signal transduction
negative regulation of canonical Wnt si...
positive regulation of cell growth
insulin receptor signaling pathway
response to organic cyclic compound
tissue regeneration
regulation of insulin−like growth factor ...
extracellular region
extracellular space
Golgi apparatus
endoplasmic reticulum lumen
protein binding
signaling receptor binding
insulin−like growth factor I binding
insulin−like growth factor binding
insulin−like growth factor II binding
BP
CC
MF
0
1
2
3
−log 10 p−value

CRP

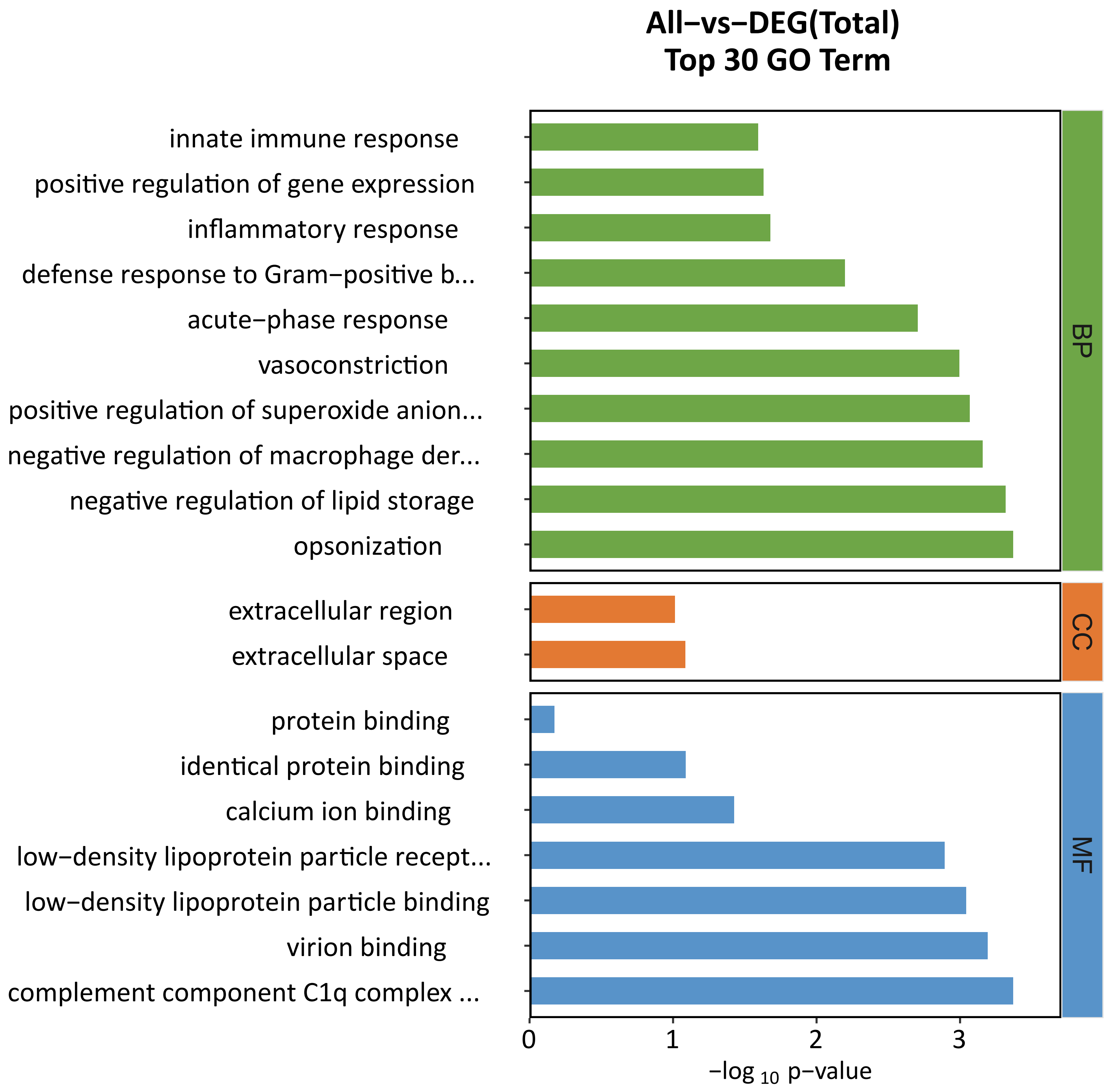
All−vs−DEG(Total)
Top 30 GO Term
innate immune response
positive regulation of gene expression
inflammatory response
defense response to Gram−positive b...
acute−phase response
vasoconstriction
positive regulation of superoxide anion...
negative regulation of macrophage der...
negative regulation of lipid storage
opsonization
extracellular region
extracellular space
protein binding
identical protein binding
calcium ion binding
low−density lipoprotein particle recept...
low−density lipoprotein particle binding
virion binding
complement component C1q complex ...
BP
CC
MF
0
1
2
3
−log 10 p−value

MPO

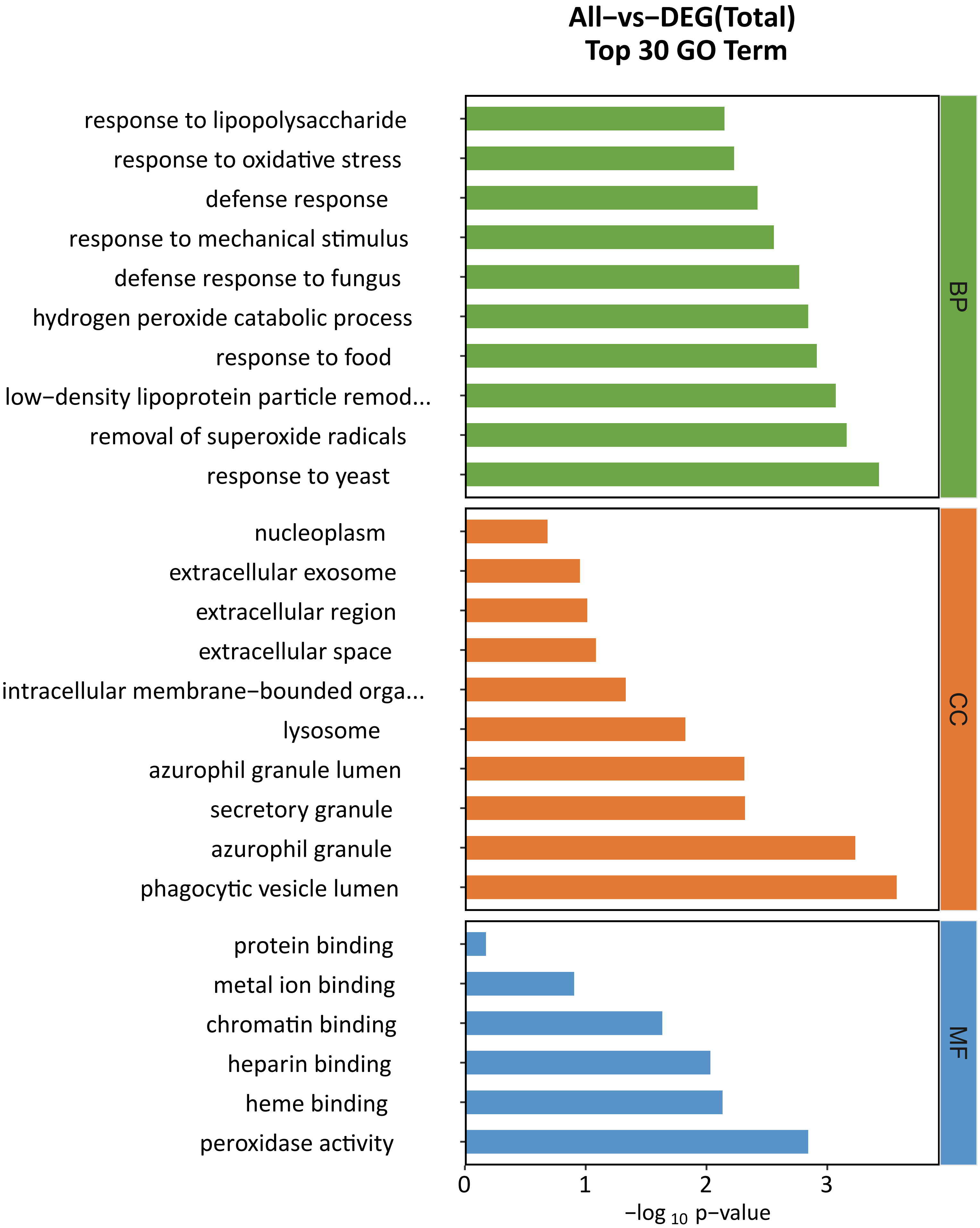
All−vs−DEG(Total)
Top 30 GO Term
response to lipopolysaccharide
response to oxidative stress
defense response
response to mechanical stimulus
defense response to fungus
hydrogen peroxide catabolic process
response to food
low−density lipoprotein particle remod...
removal of superoxide radicals
response to yeast
nucleoplasm
extracellular exosome
extracellular region
extracellular space
intracellular membrane−bounded orga...
lysosome
azurophil granule lumen
secretory granule
azurophil granule
phagocytic vesicle lumen
protein binding
metal ion binding
chromatin binding
heparin binding
heme binding
peroxidase activity
BP
CC
MF
0
1
2
3
−log 10 p−value
